# Walking indoors, walking outdoors: an fMRI study

**DOI:** 10.3389/fpsyg.2015.01502

**Published:** 2015-10-01

**Authors:** Riccardo Dalla Volta, Fabrizio Fasano, Antonio Cerasa, Graziella Mangone, Aldo Quattrone, Giovanni Buccino

**Affiliations:** ^1^Dipartimento di Scienze Mediche e Chirurgiche, Università Magna GraeciaCatanzaro, Italy; ^2^Dipartimento di Neuroscienze, Università di ParmaParma, Italy; ^3^IBFM Istituto di Bioimmagini e Fisiologia Molecolare, Consiglio Nazionale delle RicercheGermaneto, Italy; ^4^IRCCS NeuromedPozzilli, Italy

**Keywords:** walking, fMRI, mirror neuron system, space coding, rehabilitation

## Abstract

An observation/execution matching system for walking has not been assessed yet. The present fMRI study was aimed at assessing whether, as for object-directed actions, an observation/execution matching system is active for walking and whether the spatial context of walking (open or narrow space) recruits different neural correlates. Two experimental conditions were employed. In the execution condition, while being scanned, participants performed walking on a rolling cylinder located just outside the scanner. The same action was performed also while observing a video presenting either an open space (a country field) or a narrow space (a corridor). In the observation condition, participants observed a video presenting an individual walking on the same cylinder on which the actual action was executed, the open space video and the narrow space video, respectively. Results showed common bilateral activations in the dorsal premotor/supplementary motor areas and in the posterior parietal lobe for both execution and observation of walking, thus supporting a matching system for this action. Moreover, specific sectors of the occipital–temporal cortex and the middle temporal gyrus were consistently active when processing a narrow space versus an open one, thus suggesting their involvement in the visuo-motor transformation required when walking in a narrow space. We forward that the present findings may have implications for rehabilitation of gait and sport training.

## Introduction

Action observation and recognition are fundamental tasks on which social interactions are based. From these abilities also derives the capacity to quickly and accurately recognize intentions and feelings of other individuals based on their non-verbal behavior. There is increasing evidence that these cognitive tasks may be explained to some extent by a mechanism matching the observed action and motor behavior with an internal motor representation of that action or motor behavior in the brain of the observer. Cortical areas endowed with this observation/execution matching mechanism (*mirror mechanism*) are known as the mirror neuron system (MNS) (Fabbri-Destro and Rizzolatti, 2008). This system has been involved in a number of cognitive functions (Hari and Kujala, 2009) including social cognition (Gallese and Goldman, 1998). In addition, this system appears to be impaired in autism spectrum disorder where social cognition is markedly affected (Iacoboni and Dapretto, 2006). It has been forwarded that the MNS embodies a unifying mechanism active whenever motor representations are recalled as during action observation, motor imagery, dreams with a motor content and so on, even in the absence of overt action (Jeannerod, 2001).

Walking is a complex motor behavior with a special relevance in social interactions (for review, see Pavlova, 2012). By observing walking, people can extract a considerable amount of information including emotional states and intentions of the agent, even from sketchy descriptions of the body segments as it occurs with point-light biological motion stimuli (for review, see Johansson et al., 1980). That said, the cortical representation of human walking is still poorly understood. Most studies in the field assessed brain activation specifically related to execution, imagery and observation of walking, taken separately. Some studies investigated brain areas common to imagery and execution or, alternatively, imagery and observation of walking. To the best of our knowledge, however, the presence of an observation/execution matching system for this action remains to be assessed. For sake of completeness, in the following paragraphs we will shortly review current literature in the field considering first studies where execution, imagery and observation of walking were taken separately. Then, we will report about studies combining two of these tasks. Finally, we will take into account studies where the motor representation of foot actions, but not specifically walking, was investigated.

During execution of walking, brain imaging studies showed activations of several cortical (medial part of primary sensory-motor cortex, pSM, supplementary motor area, SMA, and premotor cortex, PM) and subcortical (basal ganglia and cerebellar vermis) structures (Greenstein et al., 1995; Ishii et al., 1995; Fukuyama et al., 1997; Tashiro et al., 2001). In some cases, also the recruitment of occipital and associative temporo-parietal cortices was found. A brain activation pattern similar to that of walking execution was also found during pure motor imagery of walking (Malouin et al., 2003; Sacco et al., 2006; Bakker et al., 2008; Jahn et al., 2008; Wang et al., 2008; la Fougere et al., 2010; van der Meulen et al., 2012). In very recent studies, a set of parietal, frontal and temporo-occipital areas was also found during observation of walking (Abdollahi et al., 2013; Maffei et al., 2015).

Other studies combining walking execution and walking imagery (Miyai et al., 2001; la Fougere et al., 2010) showed a pattern of activation largely shared by both tasks. Differential activation between the two tasks was present in the primary motor cortex, which was typically engaged only during the actual execution of action. Motor imagery of walking has been compared also to walking observation (Iseki et al., 2008). Among common cortical structures subserving both tasks there were dorsal PM area bilaterally, left SMA and right SPL. In summary, results indicate a recruitment of the cortical sensory-motor system, with a significant convergence between execution and imagery on the one hand and imagery and observation on the other. Indeed, the notion that motor imagery and motor execution share common neural substrates is well established also for hand actions (Stephan et al., 1995; Decety, 1996; Porro et al., 1996; Gerardin et al., 2000; Solodkin et al., 2004).

It is worth stressing that for technical reasons, in all studies mentioned above where PET was employed, actual walking was an oﬄine task executed before undergoing scanning. As for fMRI studies, since walking is a motor task rather hard to be performed in a scanner, participants were asked to perform a motor imagery since imagery and actual walking execution partially share the same neural substrates.

Some studies have assessed the neural structures involved in foot and leg actions, but not specifically walking. The activation of a dorsal sector of the PM cortex and the parietal lobe has been shown during mere observation of foot actions (Buccino et al., 2001; Wheaton et al., 2004; Sakreida et al., 2005). During motor imagery of foot plantar- and/or dorsiflexion (Cramer et al., 2005, 2007; Gustin et al., 2010), activations were found in the pSM cortex and SMA but also in the cerebellum and subcortical structures (basal ganglia and thalamus). Similar activations were also found in studies where participants were asked to perform actual execution of foot actions in combination with motor imagery or observation of the same actions (Lafleur et al., 2002; Alkadhi et al., 2005; Enzinger et al., 2008; Hotz-Boendermaker et al., 2008; Orr et al., 2008; Rocca and Filippi, 2010; Yuan et al., 2010). Christensen et al. (2000) showed a similar pattern of activation during execution and imagination of bicycling.

The present fMRI study was aimed at investigating the common neural structures recruited during the execution and observation of walking. In order to study the correlates of active walking inside the MR scanner, we employed a rolling cylinder that allowed participants to move lower limbs as if they were really walking. Moreover, we were interested in assessing whether walking in different environments, namely an open space (country field) or a narrow space (corridor) may recruit different and specific neural substrates. Because of its motor relevance, in fact, there is evidence that the space near the body is differently coded from the space far from the body (Fogassi and Luppino, 2005; Brozzoli et al., 2012). At least for the hand, neurons were discovered in the monkey that preferred actions performed either near or far from the animal (Caggiano et al., 2009). Recent results by Costantini et al. (2010), suggest that perceiving affordances of an object recruits a motor act only when the object is presented within the near space of participants where interactions with objects are possible. As far as walking is concerned, behavioral and clinical evidence suggest that different space features in which walking occurs may be differently coded (Hashimoto, 2006; Salsone et al., 2009; Schicke et al., 2009).

## Materials and Methods

### Subjects

We studied 18 healthy Italian subjects (7 females, mean age 24, range 19–28 years) with no previous history of neurological or psychiatric disorders. All participants gave written informed consent, according to the Helsinki Declaration. The present study was approved by the Ethical Committee of the University “Magna Graecia” of Catanzaro.

### Stimuli

**FIGURE 1:**
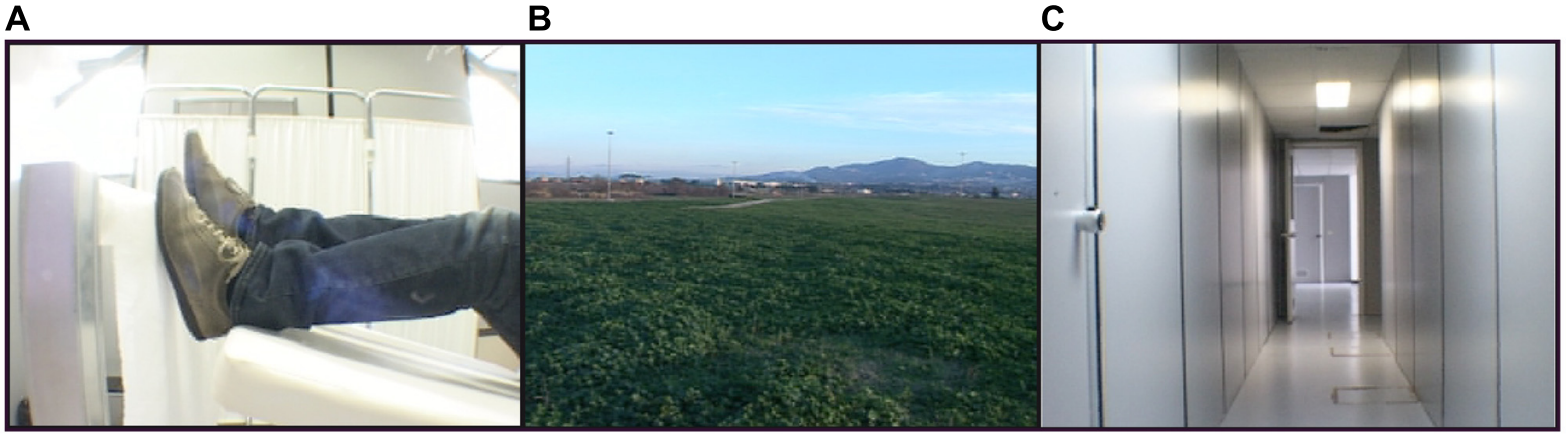
**Stimuli. (A)** A still frame taken from the video clip showing an individual lying supine and walking on a rolling cylinder. **(B)** A still frame taken from the video clip showing a countryside view (open space). **(C)** A still frame taken from the video clip showing a corridor (narrow space).

The stimuli presented in the experiment consisted of video clips depicting either a walking action or different spatial contexts (**Figure 1**). The *Walking* video clip showed the lower limbs of an individual lying supine and performing a walking action on a rolling cylinder. The *Open Space* video clip showed a countryside view while the *Narrow Space* video clip showed a narrow corridor. In both the videos depicting a space, the scene was filmed while the cameraman was actually walking in the countryside or in the corridor. In this way, the observation of these videos gave participants the feeling of walking into the observed space. Still images taken from the above mentioned video clips served as controls (*Still Walking*, *Still Open Space* and *Still Narrow Space*). Each video clip and the corresponding still image lasted 21 s and were preceded by the presentation (3 s) of written words at the center of the screen in order to cue participants.

### Experimental Procedure

**FIGURE 2:**
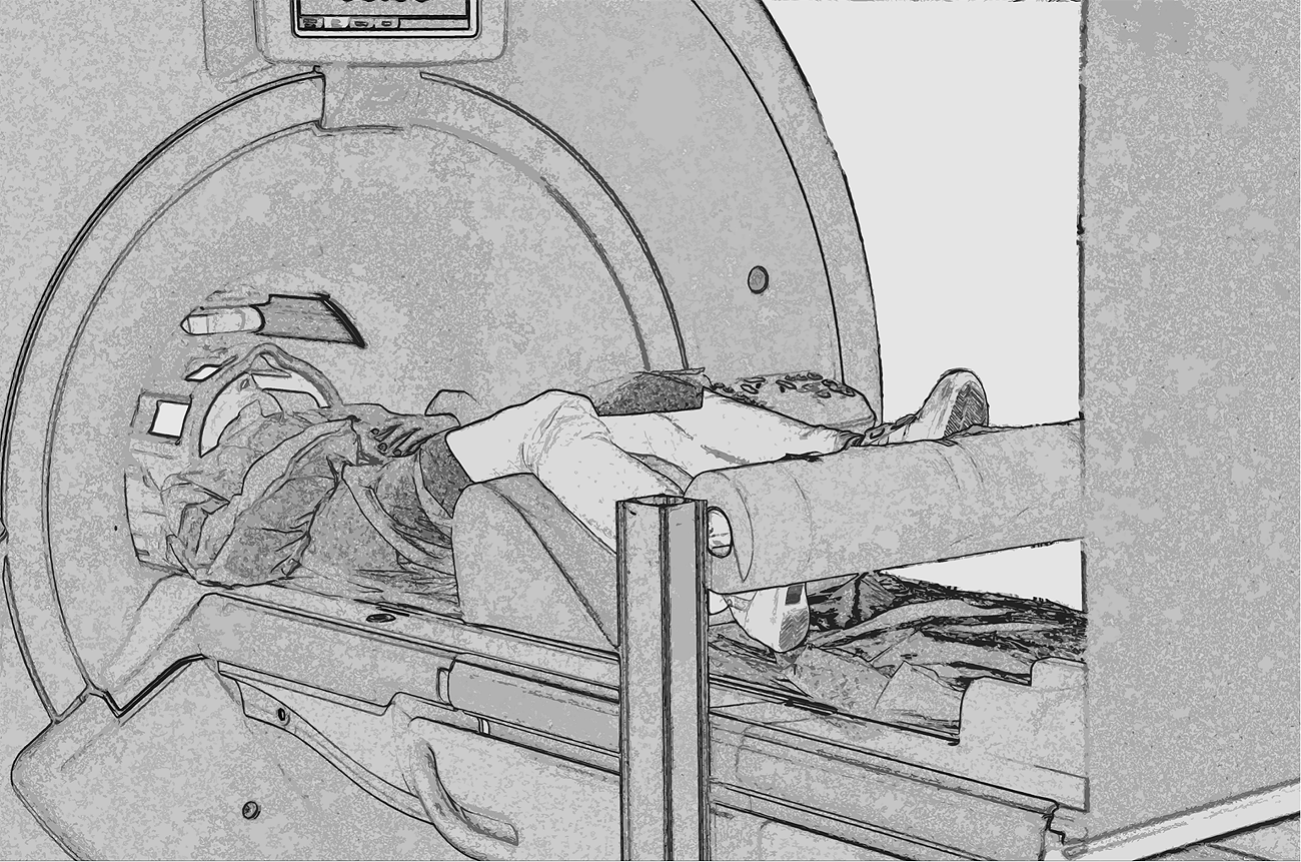
**Experimental set up**. Picture showing a participant walking on the rolling cylinder while being scanned.

Each participant comfortably lay in the scanner with a forehead restraining strip and foam pads to ensure head fixation and minimize motion during scanning. Moreover, an adhesive band was fixed on the participant’s jaw to help control movement. Visual stimuli were projected on an acrylic screen inside the MRI room. A mirror was placed on the head coil at 45° to the screen and the participant’s line of sight. Functional MRI timing parameters and triggering of the visual stimulation were performed by an in-house software developed in LabView (National Instruments, Austin, TX, USA). Before scanning, all participants completed a 10-min practice session (which included stimuli different from those presented in the scanner). A cylinder rolling around a pivot was positioned in correspondence of the feet of participants that had their legs supported by a semi-rigid wedge (**Figure 2**).

**FIGURE 3:**
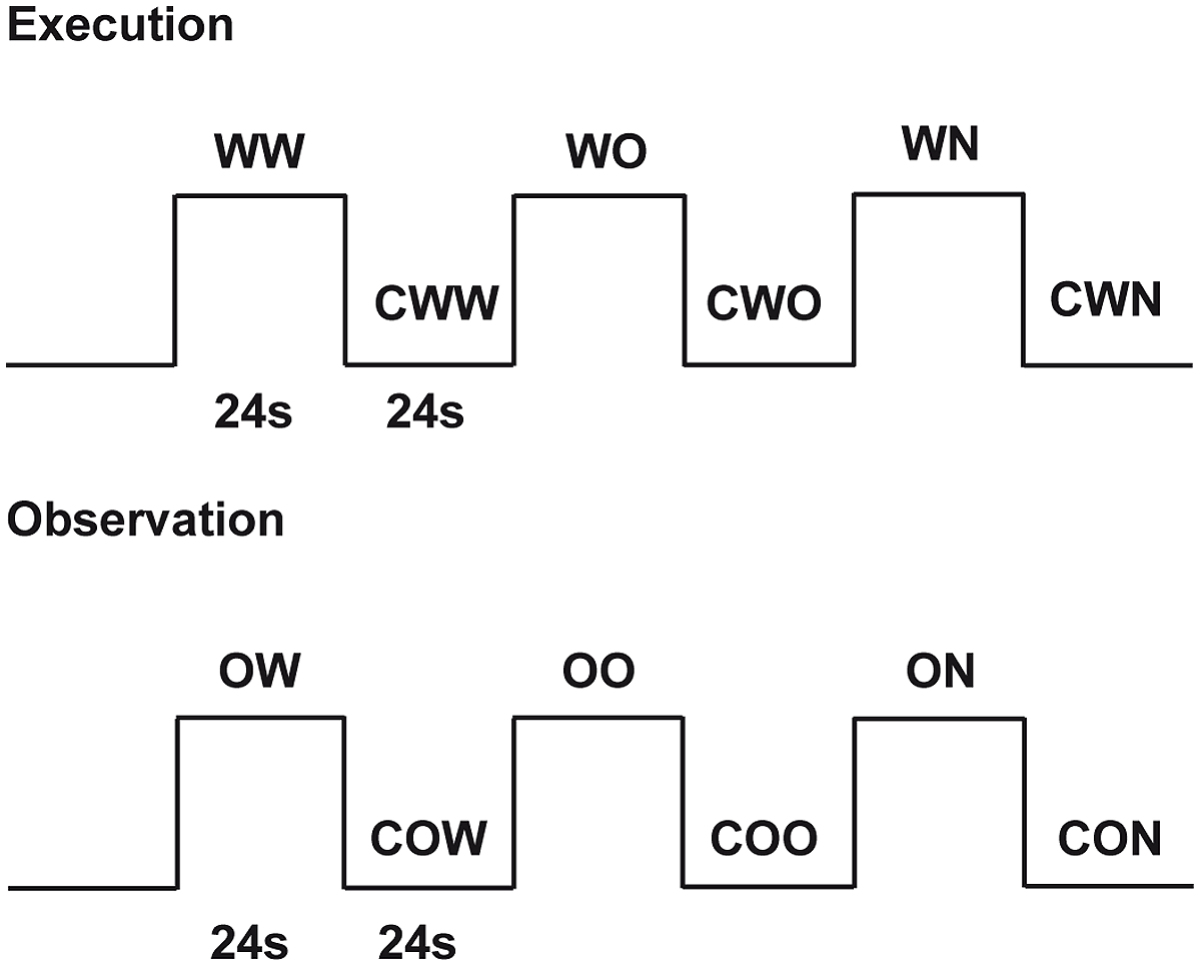
**Experimental design**. Graphic representation of the experimental design used in the present experiment. Twenty-four seconds included 3 s for the presentation of a written instruction and 21 s of task duration.

The study consisted of two experimental conditions: (1) execution and (2) observation. During execution, subjects performed three different tasks: (a) walking on a rolling cylinder while looking at a gray screen (*Walking*, WW), (b) walking on a rolling cylinder while looking at the *Open Space* video (*Open Space Walking*, WO) and (c) walking on a rolling cylinder while looking at the *Narrow Space* video (*Narrow Space Walking*, WN). For each of these tasks, we used the following as controls: (a) gently pressing the rolling cylinder with the feet while looking at a gray screen (*Control for Walking*, CWW), (b) gently pressing the rolling cylinder with the feet while looking at the *Still Open Space* (*Control for Open Space Walking*, CWO), and (c) gently pressing the rolling cylinder with the feet while looking at *Still Narrow Space* (*Control for Narrow Space Walking*, CWN). Before scanning, participants were trained to walk on the rolling cylinder and minimize head and trunk movements. During observation, participants performed the following tasks: (a) observing the *Walking* video (*Walking Observation*, OW), (b) observing the *Open Space* video (*Open Space Observation*, OO) and (c) observing the *Narrow Space* video (*Narrow Space Observation*, ON). For each of these tasks, we used the following as controls (a) observing the *Still Walking* (*Control for Walking Observation*, COW), (b) observing the *Still Open Space* (*Control for Open Space Observation*, COO), and (c) observing the *Still Narrow Space* (*Control for Narrow Space Observation*, CON). Each task was cued by a written instruction. The appearance of the words ‘cammina’ (i.e., walk), ‘premi’ (i.e., press), and ‘guarda’ (i.e., look at) cued participants to perform the different tasks. The present study used a block design with a pseudo-random presentation of the tasks (**Figure 3**). Each task was followed by the correspondent control (e.g., when participants walked on the rolling cylinder while looking at a gray screen, what followed was the control in which participants gently pressed the rolling cylinder with their feet while looking at the gray screen) and was presented four times.

### Data Acquisition

MR data were acquired with a 3 Tesla scanner (Discovery MR-750, General Electric, Milwaukee, WI, USA) equipped with a 32-channel receiver head-coil. Functional images were acquired using a T2*-weighted gradient-echo, echo-planar (EPI) pulse sequence (acceleration factor (asset) 2, 32 interleaved transverse slices covering the whole brain, TR = 2000ms, TE = 30ms, flip-angle = 90°, FOV = 240 mm × 240mm, inter-slice gap = 0 mm, slice thickness = 4 mm, in-plane resolution 1.88 mm × 1.88 mm). From each participant, 576 volumes were collected in a single session. Additionally, a 3D structural T1-weighted spoiled gradient (SPGR) echo sequence was acquired.

### Data Analysis

Data analysis was performed with SPM8 (Statistical Parametric Mapping software by the Wellcome Trust Centre for Neuroimaging, Leopold Muller Functional Imaging Laboratory, University College of London, London, UK; http://www.fil.ion.ucl.ac.uk) running on MATLAB R2011a (The Mathworks, Inc., Natick, MA, USA). The mean EPI was first computed for each participant and visually inspected to ensure that none showed artifacts. The first four EPI volumes of each functional run were discarded to allow for T1 equilibration effects. For each subject, all volumes were spatially realigned to the first volume of the run. Next, images were normalized to the EPI SPM template, re-sampled in 2 mm × 2 mm × 2mm voxels using trilinear interpolation in space and spatially smoothed with an 8 mm full-width half-maximum isotropic Gaussian kernel for the group analysis. Two participants showing head movements greater than 2 mm were excluded from all subsequent analyses.

Data were analyzed using a random-effects model (Friston et al., 1999), implemented in a two-level procedure. In the first level, single-subject fMRI data entered an independent General Linear Model (GLM) by design-matrixes modeling the onsets and durations of 12 experimental factors, 6 related to the experimental tasks, and 6 related to their corresponding controls. For each participant, we generated contrast images displaying the effect of the experimental tasks contrasted with the respective controls: WW-CWW, WO-CWO, WN-CWN, OW-COW, OO-COO, ON-CON. In addition, images displaying the effect of walking in a narrow space contrasted with walking in an open one and vice versa, and images displaying the effect of observing a narrow space contrasted with observing an open one and vice versa were generated: WN-WO, WO-WN, ON-OO, OO-ON. Next, each contrast entered a second-level GLM to obtain: (*i*) SPM{T} maps (one sample *t*-test) related to each task at group-level and (*ii*) SPM{min(T)} maps (*conjunction* analysis) to test for (a) the existence of an observation/execution matching system for walking using the following contrasts (WW-CWW)∩(OW-COW), and (b) the existence of areas specifically involved in coding peripersonal and extrapersonal space, using the following contrasts: (WN-WO)∩(ON-OO) and (WO-WN)∩(OO-ON), respectively (Friston et al., 1999). To this aim, we performed an SPM ‘*conjunction null’* analysis (Nichols et al., 2005). Given the conservative nature of this analysis (Friston et al., 2005), we report data with a *p*-value < 0.001 uncorrected. A threshold of 10 was applied on cluster dimension. For all analyses, location of the activation foci was determined in the stereotaxic space of the MNI coordinates system. Those cerebral regions for which maps are provided were also localized with reference to cytoarchitectonical probabilistic maps of the human brain, using the SPM-Anatomy toolbox v1.7 (Eickhoff et al., 2005).

### Head Movement

Since we asked for actual execution of walking inside the scanner, we were particularly careful in evaluating motion artifacts and minimizing their impact on the results. To this aim, for each subject all volumes were realigned to the first acquired one by applying a 6-parameters (rigid body) spatial transformation computed for each volume using a least-square approach. The mean head movement parameters were: *x*-direction 0.49 mm (±0.33), *y*-direction 0.41 (±0.26), and *z*-direction 1.94 (±1.13). The estimated six spatial transformation parameters computed for each volume entered as regressors in the subsequent model design matrix to de-convolve the head movement effect from the hemodynamic response.

## Results

**FIGURE 4:**
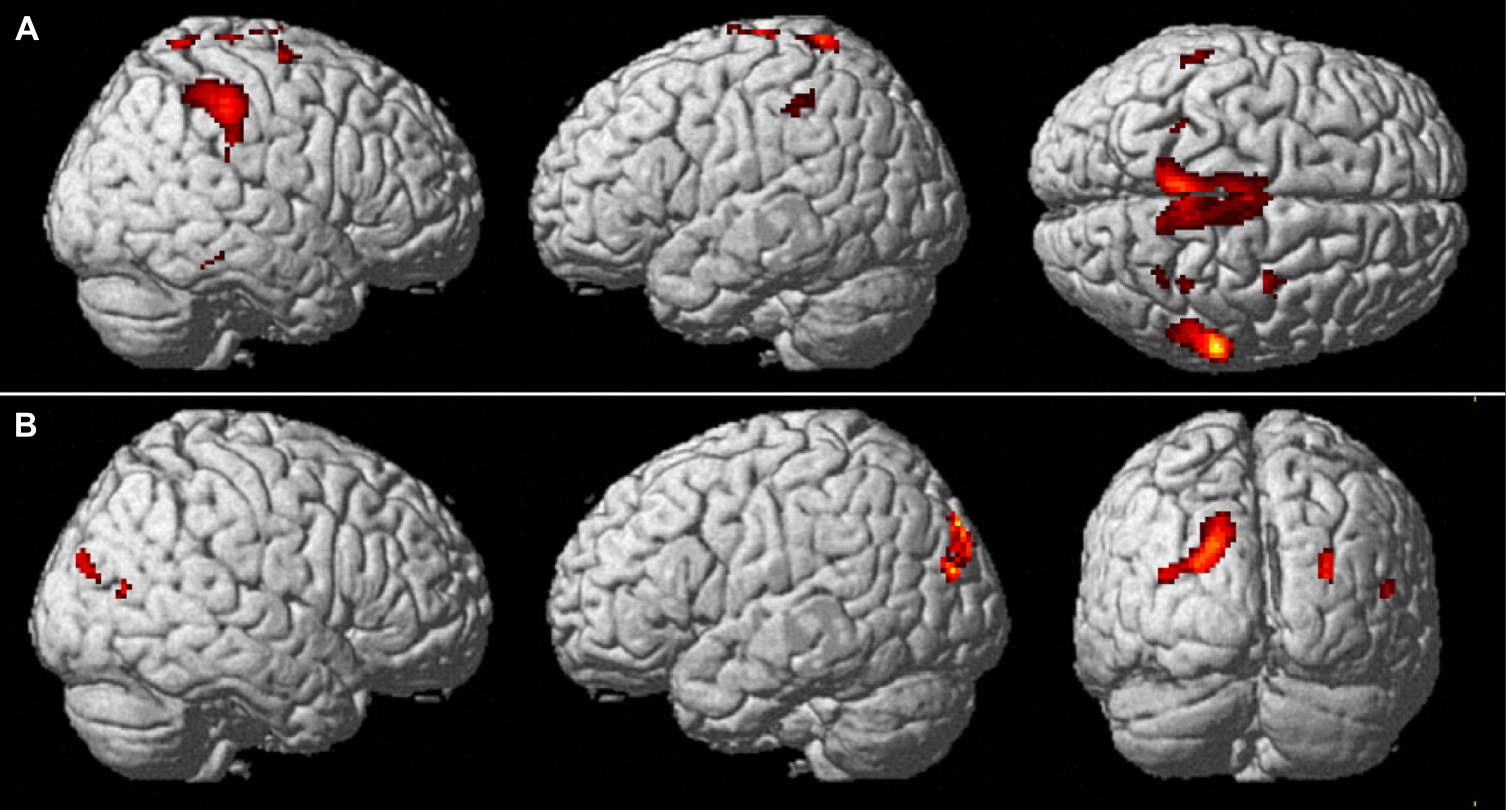
**Common brain activations from conjunction null analysis. (A)** Upper row: Activations common to walking observation and execution (*p* < 0.001, uncorrected, *k* = 10). Activity is superimposed on a rendered brain viewed from the right (left panel), the left (middle panel), and the top (right panel). **(B)** Lower row: Activations specifically related to the processing of a narrow space as compared to an open one (*p* < 0.001 uncorrected, *k* = 10). Activity is superimposed on a rendered brain viewed from the right (left panel), the left (middle panel), and the posterior (left panel).

The main results of the conjunction analyses are shown in **Figure 4**. **Table 1** lists the MNI standard brain coordinates of the local maxima of BOLD-signal increases as revealed by all conjunction analyses. Common activations for execution and observation of walking, as revealed by the conjunction analysis (WW-CWW)∩(OW-COW), are shown in **Figure 4A**. Basically, a set of parieto-frontal areas emerged. Frontal activation foci were present in SMA and extended to adjacent dorsal PM cortex, bilaterally. In the right hemisphere a distinct spot in the dorsal premotor cortex was also present. Parietal activation foci were present in right SPL and in IPL bilaterally. In addition, activation foci were present also in the cerebellar vermis and in both cerebellar hemispheres.

**Table 1:** MNI coordinates of local maxima of the activation foci (conjunction null analysis).

AnatomicalRegion	(ATB^∗^)	Left				(ATB^∗^)	Right			
		*x*	*y*	*z*	*Z*-score		*x*	*y*	*z*	*Z*-score
**(A) Walking observation/execution**										
-Supplementary Motor Area (SMA)	(6)	-2	-18	76	4.68					
-SMA		-4	-38	74	4.85		6	-38	74	4.62
-Precentral Gyrus							38	-8	66	3.67
-Superior Parietal Lobule							32	-52	72	3.43
-Inferior Parietal Lobule	(PF)	-58	-40	48	3.84	(PF)	60	-32	46	5.08
-Cerebellum	(Vermis)	-4	-72	–42	4.62					
-Cerebellum		-26	-46	–28	3.95		20	-36	–28	4.31
**(B)Narrow vs. open space walk**										
-IntraParietal-TransverseOccipital (IPTO)		-34	-86	18	3.62					
-IPTO	(7, 18)	-14	-90	30	3.74		28	-86	22	3.65
-IPTO		-14	-86	38	3.77					
-Middle Occipital Gyrus (MOG)						(PG)	52	-70	12	3.80
**(C)Open vs. narrow space walk**										
-Inferior Occipital Gyrus	(17)	-14	-98	-8	4.18					

***(A)** Conjunction investigating common brain activations during walking execution and walking observation.*

***(B)** and **(C)** Conjunctions which investigated walking in narrow and open space, respectively.*

*All reported local maxima were significant with *p* < 0.001 uncorrected and an extent threshold *k* = 10 voxels.*

*^∗^ATB: most probable anatomical region in the Anatomy Toolbox 1.7, as reported by Eickhoff et al., 2005*.

Cerebral activations related to the processing of a narrow space with respect to an open one, as revealed by the conjunction analysis (WN-WO)∩(ON-OO), are shown in **Figure 4B**. Basically, a set of occipital and parietal areas emerged. In particular, an occipital activation focus was located in the right middle occipital gyrus (MOG). The most caudal part of the intraparietal sulcus intersecting the transverse occipital sulcus (IntraParietal-TransverseOccipital, IPTO) was active bilaterally, but more largely represented in the left hemisphere where it extended toward the SPL.

Cerebral activations related to the processing of an open space with respect to a narrow one, as assessed by the conjunction analysis (WO-WN)∩(OO-ON), were found in the left inferior occipital gyrus.

## Discussion

The present findings support the existence of an observation/execution matching system for walking and the presence of specific brain areas devoted to the coding of near space during walking. We will discuss these points eventually including their potential implication in rehabilitation of walking.

By means of an experimental setting where participants had the possibility to walk on a rolling cylinder while being scanned, we could investigate active walking, in addition to walking observation, while reducing to a minimum movement artifacts. However, it is worth underlining that walking in a scanner remains an approximation of walking in natural contexts, thus preventing us from the possibility of assessing the role of postural adjustments, gravity and so on. That said, the results of the present study revealed a set of parieto-frontal areas active during both execution and observation of walking, including the dorsal PM, SMA, IPL, and a dorsal sector of SPL. These areas therefore, may be considered part of a wider system recruited during both execution and observation of actions performed with different biological effectors (MNS, Rizzolatti and Craighero, 2004; Fabbri-Destro and Rizzolatti, 2008; Hari and Kujala, 2009). So far, in the monkey there is no evidence of an observation/execution matching system specific for lower limb actions including walking while a mirror mechanism has been described for hand or mouth actions (Gallese et al., 1996; Rizzolatti et al., 1996; Ferrari et al., 2003; Rizzolatti and Craighero, 2004). As for humans, several imaging studies suggest that the MNS is not restricted to hand- and mouth-related actions but it extends also to cover foot actions (Buccino et al., 2001; Wheaton et al., 2004; Sakreida et al., 2005). The present data extend the MNS also to walking and are in line with previous studies assessing the neural substrates of walking imagery (Malouin et al., 2003; Sacco et al., 2006; Bakker et al., 2008; Jahn et al., 2008; Wang et al., 2008; la Fougere et al., 2010; van der Meulen et al., 2012). As a whole, they further support the notion that action execution, action observation and motor imagery share common neural structures (Jeannerod, 2001).

The frontal nodes of the MNS for walking are represented by the SMA and the dorsal PM. Previous studies reported that rostral SMA is particularly active in planning spatiotemporal aspects of action and in updating motor plans for temporally ordered subsequent movements (Roland et al., 1980; Tanji and Kurata, 1985; Shibasaki et al., 1993). The recruitment of SMA in the present study suggests a role for this area in providing proper sequencing and timing of limb movements during actual walking. Dorsal PM is endowed with a motor representation of lower limbs (Kurata, 1989; Godschalk et al., 1995) and a role of this region in locomotor control has long been suggested (Freund and Hummelsheim, 1985). Dorsal PM, therefore, may exert the control of walking especially when it is guided by visual information. In the right hemisphere there also was a distinct dorsal premotor spot that largely coincides with the one described by Buccino et al. (2001) during the observation of foot actions, either object- or non-object-directed.

The posterior nodes of the MNS for walking are represented by sectors of the posterior parietal cortex. The dorsal portion of SPL is involved in integrating proprioceptive information related to the current body position into a motor plan (Andersen et al., 1997; de Lange et al., 2006) and in combining visual and somatosensory information in order to guide spatially directed movements (Andersen et al., 1997; Wenderoth et al., 2006). During walking execution, SPL activation may be related to the processing of visual and somatosensory feedback. During walking observation the same functional activation may represent a re-enactment of the sensory aspects of the observed action. Indeed a mirror mechanism has been described also for sensory information (Keysers et al., 2004; Ebisch et al., 2008; Gazzola and Keysers, 2009). It has been proposed that actual and imagined movements involve prediction of the sensory consequences of the action (Wolpert et al., 1998; Blakemore and Sirigu, 2003). This may be true also for observed actions. Bakker et al. (2008) reported activation of a dorsomedial sector of SPL closely corresponding to that of the present study when participants performed a motor imagery of walking along a narrow path compared to a broad path. The authors interpreted this finding as indicating that during motor imagery sensory information is generated in the absence of concurrent action production.

As for IPL, this cortical sector has long been involved in coding the pragmatic features of an object that are relevant for a biological effector (for instance, the hand) in order to act properly upon it (Binkofski et al., 1999; Chao and Martin, 2000; Grèzes and Decety, 2002; Grèzes et al., 2003). We suggest that during walking IPL may code the interaction between foot and the surface on which individuals are requested to walk. In other words, this region may provide information on pragmatic features of the surface (for instance, the presence of holes or bumps) relevant for walking properly on it. It is noteworthy that in the present study while actually walking, participants had to interact with a rolling cylinder. In previous studies IPL has been shown to be involved in motor imagery (Malouin et al., 2003) and observation of walking (Iseki et al., 2008).

During both observation and execution of walking, we found a set of activation foci also in the cerebellum which included the vermis and both cerebellar hemispheres. Cerebellar activation during execution and imagery of walking (Fukuyama et al., 1997; la Fougere et al., 2010; van der Meulen et al., 2012) as well as during lower limb movements (Rocca and Filippi, 2010) was previously found. Despite the cerebellum is not considered as a node of the MNS, it might come into play whenever motor sequences such as walking are executed and/or observed (Molinari et al., 2008).

As far as space coding is concerned, our findings show that there are specific regions in the brain involved in the coding of a narrow space as compared to an open one. When considering hand-object interactions, pivotal electrophysiological studies in the monkey showed the existence of bimodal visual and tactile neurons that discharge when the objects are within a reachable distance. Such neurons have been identified in several regions of the monkey brain, including PM and parietal areas, and it has been forwarded that they code for a near space where it is possible for individuals to interact with objects (Rizzolatti et al., 1997; Fogassi et al., 1999; Graziano, 2001). This space has been called *peripersonal* space to distinguish it from a far space irrelevant for action execution (extrapersonal space). In humans, brain imaging studies have confirmed the presence of a parieto-frontal circuit coding for peripersonal space around the face and hands (Bremmer et al., 2001; Sereno and Huang, 2006; Makin et al., 2007), with additional areas centered in superior parieto-occipital cortex (Quinlan and Culham, 2007; Gallivan et al., 2009) and lateral occipital cortex (Makin et al., 2007). In keeping with fMRI studies, behavioral studies have shown a clear distinction between a peripersonal and an extrapersonal space (for review see Spence et al., 2004, 2008; Brozzoli et al., 2012). All these studies focused on face/hand-object interactions in peripersonal space while less evidence is available for the coding of a motorically relevant space for foot actions. There is a suggestion that a peripersonal space representation would seem to be an efficient organizational principle not only for the upper but also for the lower limb (Graziano et al., 2002; Graziano and Cooke, 2006). In a behavioral study, for instance, Schicke et al. (2009) employed a cross modal congruency task and found that congruency effects did not differ between hand and foot suggesting a representation of peripersonal space also around the feet. It is worth stressing that in our study, when participants were required to observe a narrow space or to walk in it, they perceived this space as peripersonal since the different elements in the seen environment (for instance, the walls of the corridor) were at a reachable distance. In contrast, when participants were required to observe an open space or to walk in it, they perceived this space as extrapersonal since the different elements (for instance, trees and hills in the background) were perceived as too far to interact with them. In the present study, when participants had to process a near space, specific activations were found in the right MOG and in the IPTO bilaterally, but more largely represented in the left hemisphere.

The right MOG corresponds to the area found active by Bakker et al. (2008) in the imagery of walking along a narrow path as compared to a broad one. This area is close to the lateral occipital cortex labeled as *extrastriate body area* (EBA) by Downing et al. (2001) that is recruited during the observation of different body parts even when they imply little motion. Bakker et al. (2008) suggested that MOG activity reflects the generation of accurate predictions of the sensory (presumably visual) consequences of a specific motor plan. As in Bakker et al. (2008), in both space videos our participants did not observe any body parts so the explanation provided by these authors may also fit our data. In more general terms, we suggest that this activation may call for a visual description of the position of body parts including foot in our particular walking set. This interpretation is well supported by the fact that in the present experiment MOG activity appears stronger in the narrow space as compared to an open one. Indeed, in the narrow space a more accurate visual description of one’s own body parts is a key requirement to interact properly with the environment. This means that extrastriate regions might be involved in generating visual imagery relevant to motor control (Toni et al., 2002; Astafiev et al., 2004; Helmich et al., 2007), as reported in other sensory domains (Blakemore and Sirigu, 2003).

As for the IPTO, Makin et al. (2007) showed a greater activation of this region in all conditions in which an object moved toward the participant’s body (near space) independent of the fact that a biological effector will interact with the object or not. Activation of the IPTO was also found in a PET study by Binkofski et al. (2003) when participants had to reach for a virtual object in a mirror positioned in front of the observer. Since this was a PET study, these findings are not fully comparable with the present ones. Nevertheless, it is worth stressing that also in the condition described by Binkofski et al. (2003) the virtual object was perceived in the near space of participants. Moreover, according to the coordinates provided by Pisella et al. (2009), IPTO is part of a region potentially damaged in patients with optic ataxia (OA). Classically, OA is considered a disorder of reaching objects presented in peripheral vision following an impairment in visuomotor integration. Since IPTO appears to code for a near space as distinct from a far one, it is reasonable that a lesion of this area may contribute to the clinical picture of OA patients. Altogether, our findings and those of the literature reviewed so far strongly suggest a specific role for IPTO in distinguishing a near space from an open and far one. As Makin et al. (2007) found this sector during a task involving the hand while in the present study we consider a task specifically related to foot actions, it is most likely that the representation of near space coded in IPTO is independent of a specific biological effector and may constitute a preliminary processing of space subserving any further visuomotor transformation involving objects located in it.

Greater activation during processing the open space as compared to the narrow one was found in the primary visual cortex. The most likely explanation for this finding is that the landscape depicted in the open space video was full of different elements (grass, road, houses, street lamp, mountains, and so on) which made the scene more vivid and visually complex than the one depicted in the narrow space video.

In our opinion the present findings may have implications in the field of rehabilitation. Indeed, they show that observing walking actions, by triggering the MNS, is effective in recruiting sectors of the cortical motor system involved in the execution of the same motor tasks. As mentioned above, it has been shown that the motor system and in particular the MNS is involved in motor execution, action observation and motor imagery. For several years, motor imagery has been used in the rehabilitation practice and sports (Mulder, 2007). The recruitment of motor representations, driven by motor imagery, can improve the quality of motor performance, even in the absence of an actual execution of action. It has been proposed (Buccino et al., 2006; Small et al., 2013) that, similarly to motor imagery, the careful observation of actions made in an ecological context may be a valid approach in rehabilitation (action observation treatment, AOT), since even action observation has proven to be effective in recruiting the motor representations of the observed actions. During AOT, patients are required to carefully observe and soon afterward execute different daily actions presented through video-clips (Small et al., 2013; Buccino, 2014). So far, AOT has been successfully applied to chronic stroke patients (Ertelt et al., 2007) and to the recovery of daily activities in PD patients (Buccino et al., 2011). Recently, a case-control study has been conducted on the efficacy of AOT in children affected by cerebral palsy (Buccino et al., 2012).

It is worth stressing that AOT has been shown to be effective also in the rehabilitation of lower limbs motor function. In detail, Pelosin et al. (2010) used AOT in the recovery of walking ability in PD patients with freezing of gait and Bellelli et al. (2010) in the rehabilitation of orthopedic patients that had undergone hip or knee replacement surgery. In our opinion, the findings of the present study, by showing the existence of an observation/execution matching system for lower limbs actions including walking, provide neurophysiological basis for this clinical evidence.

Furthermore, action observation of lower limbs actions has the potential to be exploited also in a variety of fields including educational activities and sport. In fact, to the best of our knowledge, while motor imagery has been used (for review, see Mulder, 2007) as a training strategy for athletes, even at competitive levels, the potential of AOT in this respect has never been systematically investigated.

## Conflict of Interest Statement

The authors declare that the research was conducted in the absence of any commercial or financial relationships that could be construed as a potential conflict of interest.
